# Supplementary material for: Individual retrotransposon integrants are differentially controlled by KZFP/KAP1-dependent histone methylation, DNA methylation and TET-mediated hydroxymethylation in naïve embryonic stem cells
Source: Epigenetics Chromatin. 2018 Feb 26;11:7. doi: 10.1186/s13072-018-0177-1 (PMC6389204; doi:10.1186/s13072-018-0177-1)
Supplement: Supplementary file 11 — Additional file 11. Pattern analysis. [file 13072_2018_177_MOESM11_ESM.zip › Patterns analysis/DataTables/extensions/FixedHeader/examples/top_left_right.html]

FixedHeader example - Header, left and right all fixed


# FixedHeader example Header, left and right all fixed

FixedHeader provides the ability to fix in place the header, footer, left and right columns of the
table. These are controlled by the options:

- `top` - default true
- `bottom` - default false
- `left` - default false
- `right` - default false

This example shows top, left and right enabled with index columns on the left and right.

Note that in such a situation as this, the FixedColumns extension might be more useful,
particularly if you want to use the scrolling options built into DataTables.


- Javascript
- HTML
- CSS
- Ajax
- Server-side script

The Javascript shown below is used to initialise the table shown in this
example:

`$(document).ready(function() {
var table = $('#example').DataTable( {
"order": [ 1, 'asc' ],
"ajax": "../../../examples/ajax/data/objects.txt",
"columns": [
{ title: '', data: null, defaultContent: "" },
{ title: 'Name', data: "name" },
{ title: 'Position', data: "position" },
{ title: 'Office', data: "office" },
{ title: 'Extn.', data: "extn" },
{ title: 'Start date', data: "start_date" },
{ title: 'Salary', data: "salary" },
{ title: '', data: null, defaultContent: "" }
],
initComplete: function () {
new $.fn.dataTable.FixedHeader( table, {
left: true,
right: true
} );
}
} );
table.on( 'order.dt search.dt', function () {
table.column(0, {search:'applied', order:'applied'}).nodes().each( function (cell, i) {
cell.innerHTML = i+1;
} );
table.column(-1, {search:'applied', order:'applied'}).nodes().each( function (cell, i) {
cell.innerHTML = i+1;
} );
} ).draw();
} );`

In addition to the above code, the following Javascript library files are loaded for use in this
example:

- ../../../media/js/jquery.js
- ../../../media/js/jquery.dataTables.js
- ../js/dataTables.fixedHeader.js

The HTML shown below is the raw HTML table element, before it has been enhanced by
DataTables:

This example uses a little bit of additional CSS beyond what is loaded from the library
files (below), in order to correctly display the table. The additional CSS used is shown
below:

`div.dataTables_wrapper {
width: 150%;
}
div.FixedHeader_Cloned.fixedLeft tbody td {
border-right: 1px solid black;
}
div.FixedHeader_Cloned.fixedRight tbody td {
border-left: 1px solid black;
}`

The following CSS library files are loaded for use in this example to provide the styling of the
table:

- ../../../media/css/jquery.dataTables.css
- ../css/dataTables.fixedHeader.css

This table loads data by Ajax. The latest data that has been loaded is shown below. This data
will update automatically as any additional data is loaded.

The script used to perform the server-side processing for this table is shown below. Please note
that this is just an example script using PHP. Server-side processing scripts can be written in any
language, using the protocol described in the
DataTables documentation.

## Other examples

### Examples

- Basic initialisation
- Header and footer fixed
- Header, left and right all
  fixed
- Multiple tables
- z-index order control

Please refer to the DataTables documentation for full
information about its API properties and methods.  
Additionally, there are a wide range of extras and
plug-ins which extend the capabilities of
DataTables.

DataTables designed and created by SpryMedia Ltd © 2007-2014  
DataTables is licensed under the MIT license.
